# Clinical characteristics, pathogen detection, and severity markers among children with acute respiratory infections during the COVID-19 restriction period: a retrospective hospital-based study

**DOI:** 10.3389/fped.2026.1893727

**Published:** 2026-09-29

**Authors:** Qiuxiang Xu, Zhuanzhuan Li, Huamei Zhao

**Affiliations:** Department of Pediatric Emergency, Shanxi Provincial Children's Hospital, Taiyuan, Shanxi Province, China

**Keywords:** acute respiratory infection, children, COVID-19, hospital-based study, mycoplasma pneumoniae, non-pharmaceutical interventions, respiratory viruses, rhinovirus

## Abstract

**Background:**

The epidemiology of pediatric acute respiratory infections (ARIs) changed substantially during the COVID-19 (coronavirus disease 2019) pandemic, largely because of school closures, masking, reduced travel, and other non-pharmaceutical interventions (NPIs). However, hospital-based data from the restriction period remain useful for describing pathogen detection patterns, clinical severity, and markers of adverse clinical course.

**Methods:**

We conducted a retrospective hospital-based study of 62 children aged <18 years who were evaluated for ARI symptoms and underwent real-time fluorescent quantitative nucleic acid testing for common respiratory pathogens, including respiratory viruses and *Mycoplasma pneumoniae*. Demographic, clinical, laboratory, treatment, severity, and outcome variables were extracted from medical records. Continuous variables were summarized using medians and ranges, and group comparisons used non-parametric tests. Given the small sample size and sparse severe-outcome events, the study was not powered to detect small effect sizes, and regression findings were interpreted conservatively as hypothesis-generating rather than confirmatory.

**Results:**

The median age was 9 years (range, 1–17), and 33 patients (53.2%) were female. Most children had mild disease (37/62, 59.7%); 19 (30.6%) had moderate disease and 6 (9.7%) had severe disease. Intensive care unit (ICU) admission was recorded in 6 patients (9.7%), and mechanical ventilatory or noninvasive respiratory support in 9 (14.5%). Fever (43.5%) and cough (22.6%) were the most frequent presenting symptoms. Median oxygen saturation was 94.7% (range, 87.2–97.8). Respiratory virus detection was documented in 15 children (24.2%). Human rhinovirus was detected in 15 children (24.2%), human bocavirus in 9 (14.5%), and *Mycoplasma pneumoniae* in 10 (16.1%); co-detection was recorded in 14 children (22.6%). Lower SpO2 and longer symptom duration differed significantly across severity categories (both *p* < 0.001). In exploratory logistic regression, each additional day of symptoms was associated with higher odds of hospital admission/continued inpatient management [odds ratio [OR], 1.4; 95% confidence interval [CI], 1.03–1.93; *p* = 0.022]. Sex was not significantly associated with clinical severity or laboratory parameters.

**Conclusions:**

In this small hospital-based pediatric ARI cohort during the COVID-19 restriction period, severe disease was uncommon, and oxygen saturation and symptom duration were the most clinically coherent markers of greater severity. The observed pathogen detection pattern is compatible with pandemic-era disruption of respiratory pathogen circulation, but causal conclusions about NPIs require larger denominator-based studies with pre-pandemic and post-restriction comparison groups.

## 1 Introduction

Acute respiratory infections (ARIs) remain among the most frequent causes of pediatric illness, health-care use, antimicrobial exposure, and hospitalization worldwide (1). Their clinical spectrum ranges from self-limited upper respiratory tract infections to severe lower respiratory tract disease, respiratory failure, and death, particularly among young children, children with comorbidities, and populations with limited access to timely care (2).

Pediatric ARIs are etiologically heterogeneous. Respiratory syncytial virus (RSV), rhinovirus/enterovirus, influenza viruses, parainfluenza viruses, adenovirus, human metapneumovirus, human bocavirus, seasonal coronaviruses, atypical bacteria such as *Mycoplasma pneumoniae*, and bacterial pathogens may produce overlapping clinical syndromes (3, 4). Because fever, cough, dyspnea, and inflammatory markers are not sufficiently pathogen-specific, laboratory testing is often required to characterize etiology and guide infection-control and antimicrobial decisions (5).

The COVID-19 pandemic created an unusual natural experiment in respiratory pathogen transmission. Non-pharmaceutical interventions (NPIs), including masking, hand hygiene, school closures, travel restrictions, isolation policies, and reduced social mixing, were associated in many settings with reduced circulation of several non-SARS-CoV-2 (severe acute respiratory syndrome coronavirus 2) respiratory viruses (6–8). Subsequent relaxation of restrictions was followed by altered seasonality and resurgence of pathogens such as RSV, influenza, rhinovirus, and *Mycoplasma pneumoniae* in several pediatric cohorts (9–11).

For small hospital-based datasets collected during the restriction period, the strongest contribution is not estimation of population prevalence or causal attribution to NPIs, but careful description of clinical patterns, pathogen detection, and severity markers (12). Such data can help hospitals interpret pandemic-era case mix, identify simple bedside indicators of severity, and design larger surveillance studies.

Therefore, this study aimed to describe the demographic, clinical, laboratory, treatment, and pathogen-detection characteristics of children evaluated for ARIs during 2020–2021, and to explore clinical markers associated with disease severity, hospital admission/continued inpatient management, and prognosis.

## 2 Methods

### 2.1 Study design, setting, and reporting framework

This was a retrospective hospital-based observational study conducted in the Department of Pediatric Emergency at Shanxi Provincial Children's Hospital, Taiyuan, Shanxi Province, China. The study period extended from January 2020 to December 2021, corresponding to the COVID-19 restriction period in China. The manuscript was structured to align with the Strengthening the Reporting of Observational Studies in Epidemiology (STROBE) recommendations for reporting observational studies (13). Because the dataset included only children with ARI encounters and pathogen testing, the study should be interpreted as a clinical case-series/observational cohort rather than as a population prevalence study.

### 2.2 Participants

Eligible participants were children aged <18 years with symptoms compatible with ARI, including fever, cough, sore throat, rhinorrhea, dyspnea, or shortness of breath, who underwent respiratory pathogen nucleic acid testing during the study period. A total of 96 children with ARI-compatible presentations were initially screened during 2020–2021. Exclusion criteria were: duplicate encounters for the same illness episode, incomplete core clinical records, or absence of respiratory pathogen test results. Applying these criteria led to exclusion of 34 children (12 duplicate encounters, 15 with incomplete core clinical records, and 7 without respiratory pathogen test results). The final analytic sample included 62 children. Because respiratory pathogen testing was ordered at the treating physician's discretion rather than through universal screening, children with more severe or prolonged symptoms may have been preferentially tested, and this selection pattern should be considered when interpreting pathogen-detection frequencies and their association with severity. The participant-selection process is summarized in the flow diagram (Figure 1).

**Figure 1:**
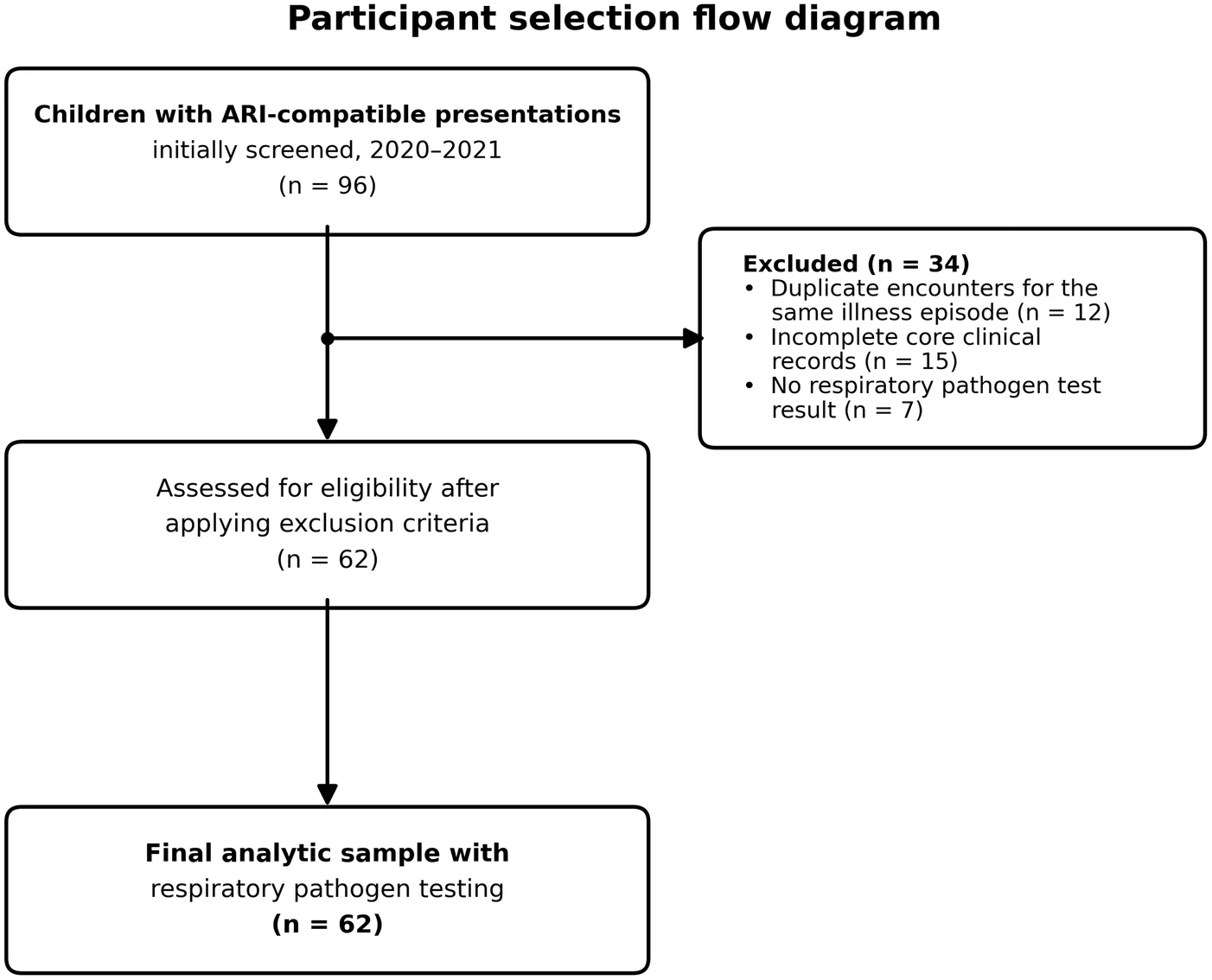
Participant selection flow diagram showing the number of children screened for eligibility, excluded (with reasons), and included in the final analytic sample.

### 2.3 Data collection

Demographic and clinical data were extracted from charts and electronic medical records by trained reviewers. Variables included age, sex, body weight, height, body mass index (BMI), symptom type and duration, oxygen saturation (SpO2), hospital admission/continued inpatient management, intensive care unit (ICU) admission, mechanical ventilation/noninvasive respiratory support, length of hospital and ICU stay, white blood cell count (WBC), C-reactive protein (CRP), procalcitonin, pathogen test results, co-detection, antiviral therapy, antibiotic therapy, corticosteroid use, supportive treatment, severity classification, and clinical outcome. Personal identifiers were coded before analysis.

### 2.4 Pathogen testing

Respiratory specimens were tested using real-time fluorescent quantitative PCR (polymerase chain reaction) assays for common respiratory viruses and *Mycoplasma pneumoniae* RNA/DNA. Nasopharyngeal swab specimens were collected within 24 h of presentation and processed in the hospital's clinical microbiology laboratory using a multiplex respiratory pathogen panel targeting influenza A and B viruses, respiratory syncytial virus, human rhinovirus, human bocavirus, human metapneumovirus, parainfluenza viruses 1–3, adenovirus, seasonal coronaviruses, and *Mycoplasma pneumoniae*. Each assay run included a positive control, a negative (no-template) control, and an internal amplification control to monitor extraction and amplification efficiency, following the reagent manufacturer's instructions and routine laboratory quality-assurance procedures. A specimen was classified as positive for a given pathogen when the cycle-threshold value was below the manufacturer-specified cutoff with a typical sigmoidal amplification curve and a valid internal control; results with an invalid internal control or atypical amplification curve were repeated before being reported.

### 2.5 Definitions

ARI was defined clinically by compatible acute respiratory symptoms. Severity was categorized as mild, moderate, or severe. Co-detection was defined as detection of more than one respiratory pathogen in the same illness episode. Clinical outcome was categorized as recovery, complication, or death according to discharge records. Hospital admission/continued inpatient management was defined as continued observation and treatment beyond the initial emergency evaluation, consistent with the emergency-department-based case mix of this cohort, in which most children were evaluated, treated, and discharged without formal ward admission. Mechanical ventilatory/noninvasive respiratory support was recorded independently of formal admission status and could be provided during emergency-department management; therefore, the number of children receiving such support does not necessarily correspond to the number meeting admission criteria.

### 2.6 Ethical considerations

**T**he study was approved by the Ethics Committee. Because this was a retrospective analysis of de-identified records, the requirement for individual informed consent was waived.

### 2.7 Statistical analysis

Continuous variables were summarized using medians and ranges because the distributions were non-normal and the sample was small. Categorical variables were summarized as frequencies and percentages. Sex-based and pathogen-detection group comparisons were performed using Mann–Whitney U tests for continuous variables and Fisher exact or chi-square tests for categorical variables as appropriate. Comparisons across severity categories used Kruskal–Wallis tests with *post-hoc* pairwise comparisons when the global test was significant. Binary logistic regression was used exploratorily to assess predictors of hospital admission/continued inpatient management. Ordinal logistic regression was used exploratorily for severity. Candidate predictors for the multivariable models (age, sex, weight, SpO2, and duration of symptoms) were selected *a priori* on the basis of clinical plausibility and were retained for multivariable modeling if they showed an association with the outcome at *p* < 0.20 in univariable analysis; because of the limited number of severe and admitted cases, each multivariable model was restricted to a maximum of two predictors to maintain an events-per-variable ratio of at least 3, and full model estimates with 95% confidence intervals for every predictor are reported in Table 1. Multicollinearity among candidate predictors was assessed using variance inflation factors, all of which were below 2.5, indicating no substantial collinearity. Model fit for the binary logistic regression was assessed using the Hosmer-Lemeshow goodness-of-fit test (chi-square=6.1, df = 8, *p* = 0.64) and Nagelkerke pseudo-R2 (0.29). For the ordinal logistic regression, the proportional odds assumption was assessed using a test of parallel lines, which was not significant (chi-square=4.8, df = 4, *p* = 0.31), supporting use of a single set of coefficients across severity categories. Because of sparse outcome cells, multinomial outcome regression was not retained as a primary inferential model; prognosis was interpreted descriptively. All *p*-values were two-sided, and *p* < 0.05 was considered statistically significant. Analyses were conducted using IBM SPSS Statistics version 27 (14). Given the small sample size, regression estimates are presented as hypothesis-generating rather than confirmatory and should be interpreted with corresponding caution.

**Table 1:** Exploratory regression findings retained for clinical interpretation.

Outcome/model	Predictor	OR (95% CI)	*p*-value	Interpretation
Hospital admission/continued inpatient management	Duration of symptoms, per day	1.40 (1.03–1.93)	0.022	Longer symptoms were associated with higher odds of admission/continued inpatient care
Hospital admission/continued inpatient management	SpO2, per 1% increase	0.58 (0.32–1.04)	0.068	Inverse association; not statistically significant
Disease severity	Age, per year	0.74 (0.54–1.02)	0.062	Borderline inverse association; not statistically significant
Disease severity	Weight, per kg	1.14 (1.04–1.25)	0.007	Interpret cautiously because of possible collinearity with age
Disease severity	SpO2, per 1% increase	0.23 (0.11–0.48)	<0.001	Higher oxygen saturation was associated with lower odds of greater severity
Disease severity	Female sex vs. male	4.12 (0.76–22.38)	0.101	Not statistically significant

CI, confidence interval; OR, odds ratio; SpO2, oxygen saturation. Sparse-outcome multinomial regression was not retained because the estimates were unstable and not clinically interpretable.

## 3 Results

### 3.1 Patient characteristics

The study included 62 children with ARI symptoms and available pathogen testing during 2020–2021. The median age was 9 years (range, 1–17), and 33 children (53.2%) were female. Median BMI was 17.0 kg/m2. Fever was the most common presenting symptom (27/62, 43.5%), followed by cough alone (14/62, 22.6%); 12 children (19.4%) had both fever and cough, and 9 (14.5%) presented with other respiratory symptoms (Table 2; Figure 2).

**Table 2:** Baseline demographic, clinical, laboratory, pathogen-detection, and treatment characteristics of the included children.

Variable	Median or n	Range or %
Demographic characteristics		
Male sex	29	46.8
Female sex	33	53.2
Age, years	9	1–17
Weight, kg	34.68	1–70
Height, cm	129.8	75.2–183.2
BMI, kg/m2	17.01	14.9–23.8
Clinical characteristics		
Hospital admission/continued inpatient management: no	55	88.7
Hospital admission/continued inpatient management: yes	7	11.3
ICU admission: yes	6	9.7
Length of hospital stay, days	0	0–16
Length of ICU stay, days	0	0–11
Duration of symptoms, days	6.3	2–25
Fever	27	43.5
Cough	14	22.6
Both fever and cough	12	19.4
Other symptoms	9	14.5
Mild severity	37	59.7
Moderate severity	19	30.6
Severe disease	6	9.7
Mechanical ventilation/noninvasive respiratory support: yes*	9	14.5
SpO2, %	94.7	87.2–97.8
Laboratory and pathogen findings		
WBC count, ×10^9/L	5.38	1.98–16.6
CRP, mg/L	9.96	0.85–78
Procalcitonin, ng/mL	0.24	0.01–1.6
Respiratory virus detected: yes	15	24.2
Human rhinovirus detected: yes	15	24.2
Human bocavirus detected: yes	9	14.5
*Mycoplasma pneumoniae* detected: yes	10	16.1
Co-detection: yes	14	22.6
Treatment		
Supportive treatment: none recorded	53	85.5
Intravenous fluids	2	3.2
Oxygen supply	7	11.3
Antiviral treatment: yes	6	9.7
Any antibiotic treatment	47	75.8
Azithromycin	29	46.8
Ceftriaxone	9	14.5
Vancomycin	4	6.5
Piperacillin/tazobactam	5	8.0
Corticosteroid use: yes	43	69.4

Values are median (range) for continuous variables and n (%) for categorical variables. BMI, body mass index; CRP, C-reactive protein; ICU, intensive care unit; SpO2, oxygen saturation; WBC, white blood cell count.

* Includes noninvasive respiratory support (e.g., high-flow oxygen) provided during emergency-department management independent of formal hospital admission status.

**Figure 2:**
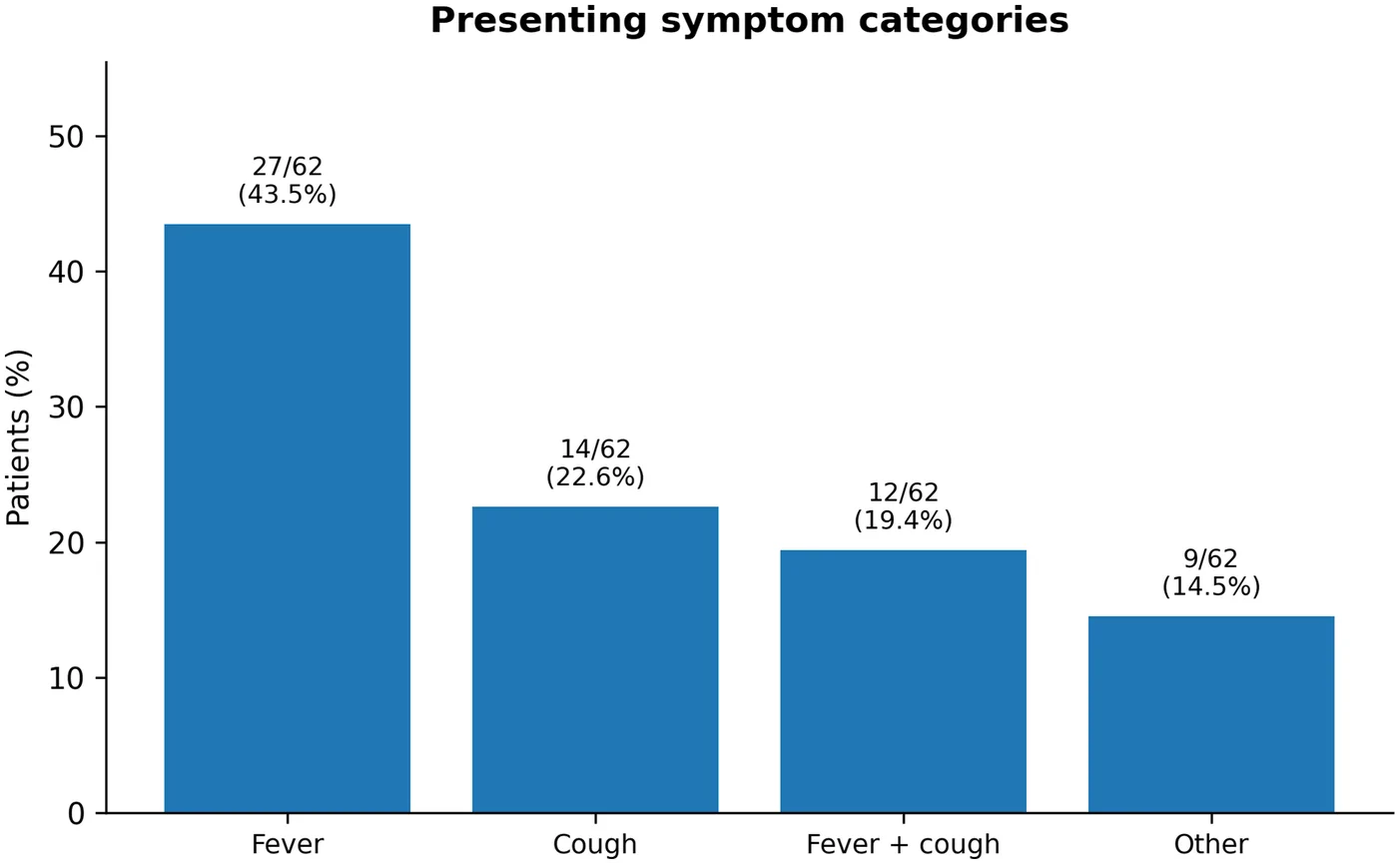
Presenting symptom categories at clinical evaluation. Categories follow the aggregate symptom classification available in the source dataset summary.

### 3.2 Clinical severity and resource use

Most children had mild disease (37/62, 59.7%), while 19 (30.6%) had moderate disease and 6 (9.7%) had severe disease (Figure 3). ICU admission was recorded in 6 children (9.7%), and mechanical ventilatory or noninvasive respiratory support was recorded in 9 children (14.5%; Table 2). Median length of hospital stay across the full cohort was 0 days (range, 0–16), and median ICU stay was 0 days (range, 0–11), reflecting the predominance of mild disease not requiring admission. Among the 7 children with hospital admission/continued inpatient management, median length of stay was 5 days (range, 1–16); among the 6 children with ICU admission, median ICU stay was 4 days (range, 1–11). Of the 9 children receiving mechanical ventilatory or noninvasive respiratory support, 7 were within the hospital-admission group, including all 6 ICU admissions, and 2 received brief noninvasive respiratory support in the emergency department without meeting formal admission criteria, which accounts for the support count exceeding the formal admission count.

**Figure 3:**
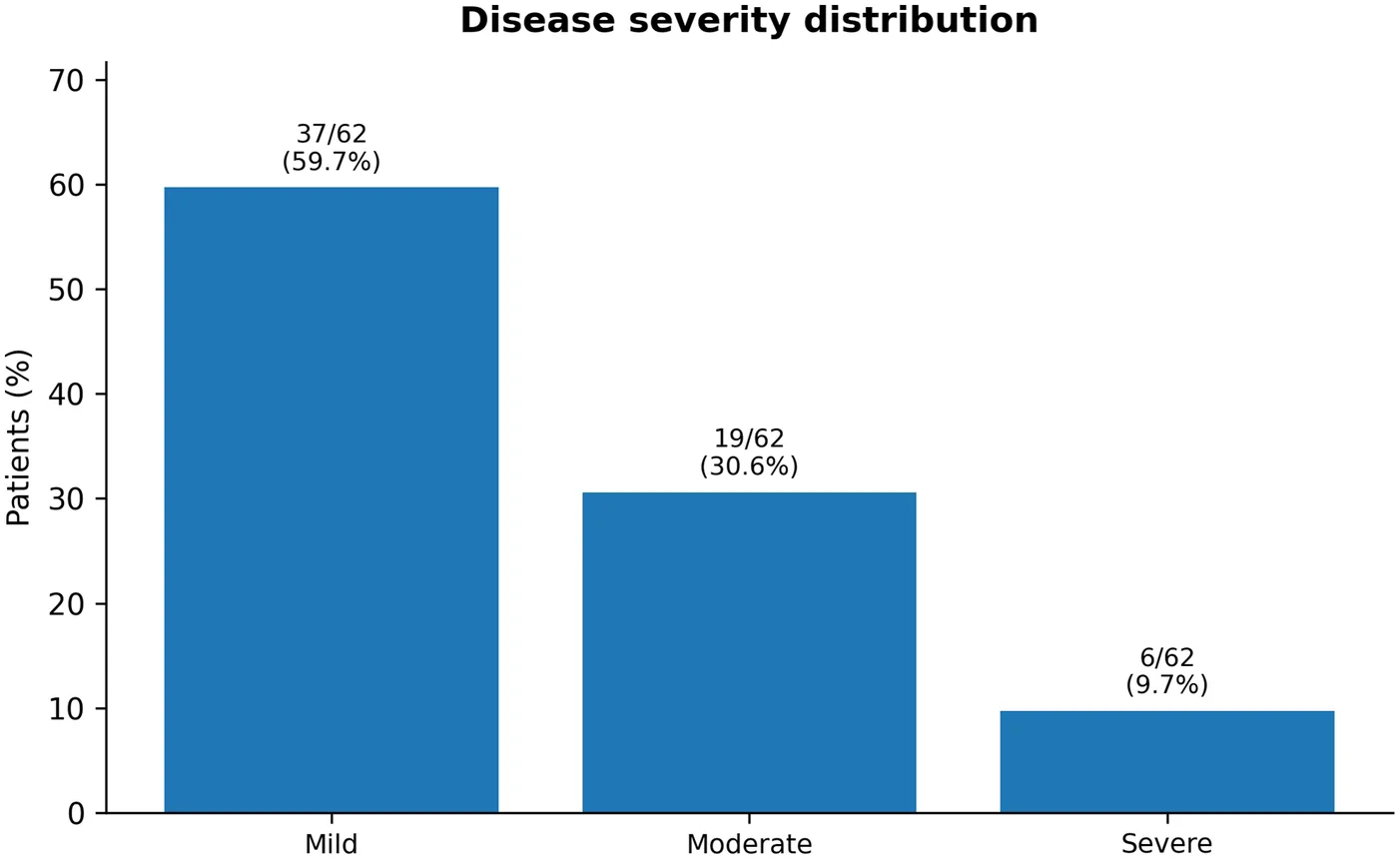
Disease severity distribution among the 62 children with acute respiratory infection symptoms and available pathogen testing during 2020-2021.

### 3.3 Laboratory findings and pathogen detection

Median SpO2 was 94.7% (range, 87.2–97.8). Median WBC count was 5.38 × 10^9/L, median CRP was 9.96 mg/L, and median procalcitonin was 0.24 ng/mL. Respiratory virus detection was recorded in 15 children (24.2%). Human rhinovirus was the most frequent detected virus (15/62, 24.2%), followed by human bocavirus (9/62, 14.5%). *Mycoplasma pneumoniae* was detected in 10 children (16.1%). Co-detection was documented in 14 children (22.6%) (Figure 4). Among the 14 co-detections, rhinovirus plus *Mycoplasma pneumoniae* accounted for 6 cases, rhinovirus plus human bocavirus for 4 cases, human bocavirus plus *Mycoplasma pneumoniae* for 3 cases, and simultaneous detection of rhinovirus, human bocavirus, and *Mycoplasma pneumoniae* was recorded in 1 case.

**Figure 4:**
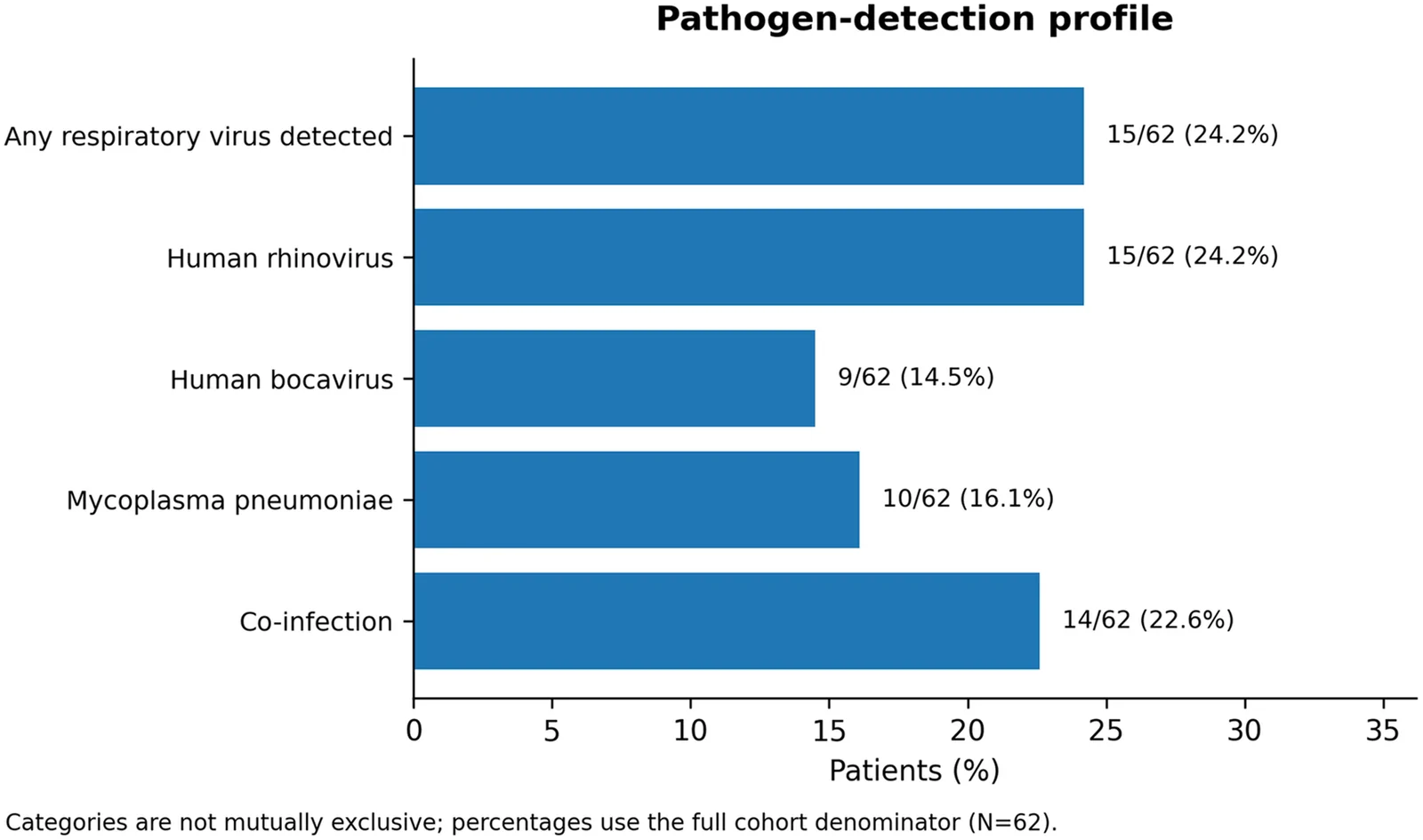
Pathogen-detection profile. Viral, *Mycoplasma pneumoniae*, and co-infection categories are not mutually exclusive; percentages are calculated using the total cohort denominator (*N* = 62).

### 3.4 Treatment patterns

Antibiotics were administered to 47 children (75.8%), most commonly azithromycin (29/62, 46.8%), followed by ceftriaxone (9/62, 14.5%), piperacillin/tazobactam (5/62, 8.0%), and vancomycin (4/62, 6.5%). Antiviral therapy was recorded in 6 children (9.7%). Corticosteroids were used in 43 children (69.4%). Oxygen supplementation was recorded in 7 children (11.3%), and intravenous fluids in 2 children (3.2%) (Figure 5). These treatment and resource-use frequencies are descriptive; formal between-severity-group statistical comparisons were not performed for these variables because of small cell counts, and the pattern should not be interpreted as evidence of severity-based indication.

**Figure 5:**
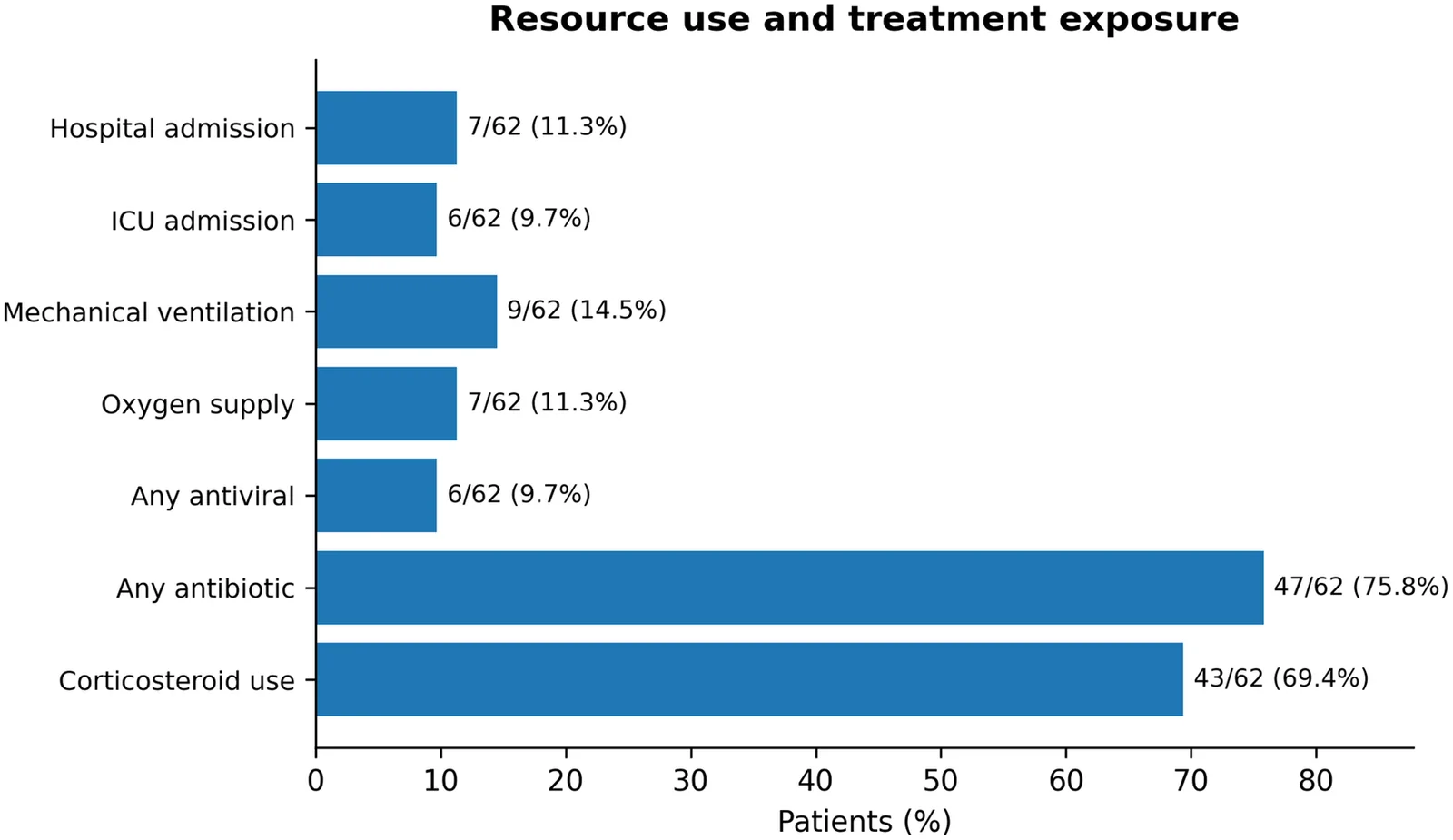
Clinical resource use and treatment exposure. Antibiotic and corticosteroid use were common despite severe disease being infrequent; interpretation requires patient-level indication data.

### 3.5 Group comparisons

No statistically significant sex-based differences were identified for symptom duration, SpO2, length of hospital stay, ICU stay, WBC count, CRP, or procalcitonin. Similarly, children with detected respiratory viruses did not differ significantly from those without detected respiratory viruses across these clinical and laboratory parameters. In severity-stratified analyses, SpO2 and symptom duration differed significantly across mild, moderate, and severe disease groups (both *p* < 0.001). *Post-hoc* comparisons indicated higher SpO2 and shorter symptom duration in mild cases compared with moderate and severe cases. Clinical outcome also differed across severity categories (*p* = 0.012). (Table 3).

**Table 3:** Severity-stratified non-parametric comparisons.

Variable	Kruskal–Wallis H	df	*p*-value	Interpretation
Age, years	0.404	2	0.817	No significant difference across severity groups
Weight, kg	0.907	2	0.636	No significant difference in unadjusted comparison
SpO2, %	33.719	2	<0.001	Lower values were associated with greater severity
Duration of symptoms, days	32.434	2	<0.001	Longer duration was associated with greater severity
Respiratory virus detected	2.742	2	0.254	No significant difference across severity groups
Co-detection	0.947	2	0.623	No significant difference across severity groups
Clinical outcome	8.888	2	0.012	Outcome differed significantly across severity groups

Pairwise *post-hoc* comparisons (Dunn's test with Bonferroni correction) were performed for SpO2 and duration of symptoms across severity groups and are summarized in the Results text. SpO2, oxygen saturation.

### 3.6 Exploratory regression findings

Because of the small number of admitted, ICU-admitted, and severe cases, these exploratory regression findings should be interpreted as hypothesis-generating rather than confirmatory. In binary logistic regression, each additional day of symptoms was associated with higher odds of hospital admission/continued inpatient management (OR, 1.4; 95% CI, 1.03–1.93; *p* = 0.022). SpO2 showed a clinically plausible inverse association with admission but did not reach conventional statistical significance (OR, 0.58; 95% CI, 0.32–1.04; *p* = 0.068). In exploratory ordinal regression for severity, lower SpO2 was independently associated with greater disease severity (OR, 0.23; 95% CI, 0.11–0.48; *p* < 0.001). Higher weight was also statistically associated with severity (OR, 1.14; 95% CI, 1.04–1.25; *p* = 0.007), although this finding should be interpreted cautiously because weight is closely related to age and may reflect residual confounding or model instability in a small sample. Age (OR, 0.74; 95% CI, 0.54–1.02; *p* = 0.062) and sex (OR for female vs. male, 4.12; 95% CI, 0.76–22.38; *p* = 0.101) were not statistically significant predictors of severity. (Table 1).

The frequency distributions summarized above for severity (Figure 3), presenting symptoms (Figure 2), pathogen detection (Figure 4), and resource use and treatment (Figure 5) reflect aggregate cohort-level percentages; between-group statistical comparisons for these categories, where formally tested, are reported in Table 3.

## 4 Discussion

In this retrospective hospital-based study of 62 children with ARIs evaluated during the COVID-19 restriction period, most cases were mild, severe disease was uncommon, and ICU admission occurred in fewer than 10% of children. The most coherent clinical severity markers were lower oxygen saturation and longer symptom duration. Respiratory virus detection was documented in approximately one quarter of children, with rhinovirus being the most frequently detected virus and *Mycoplasma pneumoniae* detected in a smaller but clinically relevant proportion. Co-detection occurred in more than one fifth of the cohort.

The finding that low SpO2 tracked with disease severity is clinically expected and strengthens internal validity. Oxygen saturation is an objective bedside indicator of lower respiratory tract involvement and impaired gas exchange. This pattern is consistent with prior pediatric respiratory cohorts in which oxygen saturation and other clinical features tracked with severity, although such bedside features alone have limited ability to identify the causative pathogen (15). Similarly, longer symptom duration may indicate delayed presentation, progressive disease, treatment failure, or more persistent infection, explaining its association with admission/continued inpatient management. Comparable associations between prolonged symptom duration and hospital-based management have been reported in rhinovirus-associated pediatric acute respiratory infection (16). These findings support prioritizing oxygen saturation and symptom trajectory in triage and follow-up decisions for pediatric ARIs.

The pathogen profile should be interpreted in the context of pandemic-era transmission disruption. Multiple studies from China and other settings have reported altered circulation, reduced activity, or delayed resurgence of several pediatric respiratory pathogens during and after COVID-19 restrictions. In mainland China, detection rates for common respiratory viruses declined during periods of strict NPIs and rebounded after restrictions were relaxed, with earlier and more pronounced rhinovirus and *Mycoplasma pneumoniae* activity reported in several post-restriction cohorts (5, 9, 10, 17–19). Comparable patterns of viral suppression followed by resurgence have also been described in Taiwan, Hong Kong, and other East Asian settings (6–8, 20). However, the present dataset cannot directly estimate the population prevalence of ARIs or quantify the independent effect of NPIs because it lacks a denominator, a pre-pandemic comparator group, and post-restriction follow-up. Therefore, the current results are best framed as a hospital-based description of ARI cases and pathogen detection during 2020–2021 rather than as evidence of NPI efficacy by themselves.

The relative predominance of rhinovirus in this cohort is biologically plausible. As a non-enveloped virus, rhinovirus is comparatively resistant to environmental degradation and to some hygiene-based interventions that primarily disrupt transmission of enveloped viruses such as influenza and respiratory syncytial virus, which may explain its continued circulation when other pathogens were suppressed (3, 21). Co-detection of rhinovirus with other respiratory pathogens is also frequently reported in pediatric cohorts and does not necessarily indicate greater severity, consistent with the absence of a significant association between virus detection and severity category in the present data (16, 21–23). *Mycoplasma pneumoniae* follows a multi-year epidemic cycle of approximately 3–7 years that is thought to reflect population immunity dynamics rather than short-term public health measures; detection of *Mycoplasma pneumoniae* in this cohort during the restriction period is therefore compatible with its intrinsic epidemic periodicity rather than a specific NPI-related effect (5, 24–26).

The high rate of antibiotic use deserves careful interpretation. Antibiotics were prescribed in three quarters of children despite respiratory virus detection in a minority and low median procalcitonin. This pattern may reflect empirical management during diagnostic uncertainty, concern for atypical pathogens such as *Mycoplasma pneumoniae*, local protocols, or severity-based treatment decisions. This proportion exceeds the confirmed bacterial or atypical-pathogen detection rate in this cohort and mirrors a broader pattern of empirical antibiotic use described in other pandemic-era pediatric respiratory cohorts (9, 19). Future studies should evaluate antimicrobial stewardship indicators, bacterial culture results, radiological findings, and outcomes to distinguish appropriate empirical therapy from potentially avoidable antibiotic exposure.

The absence of significant sex-based differences suggests that sex was not a major determinant of laboratory findings or clinical course in this small cohort. Although the original ordinal model produced a numerically higher odds ratio for female sex, this was not statistically significant and should not be interpreted as evidence of sex-related severity differences. This null finding is consistent with most pediatric ARI cohorts, in which sex has not emerged as a consistent independent predictor of severity (24, 27). Broader demographic and clinical characteristics nonetheless remain relevant to the risk and clinical presentation of respiratory infections in paediatric patients (28).

### 4.1 Strengths and limitations

This study provides real-world hospital-based data during a unique period of respiratory pathogen disruption. It includes demographic, clinical, laboratory, treatment, pathogen-detection, severity, and outcome data, allowing integrated description of clinical phenotype and severity markers.

Important limitations must be acknowledged. First, the sample size was small, and only six severe cases were recorded; therefore, multivariable models are exploratory and at risk of overfitting. Second, the single-center retrospective design limits generalizability and may introduce selection, documentation, and testing biases, including selection bias from physician-discretion pathogen testing rather than universal screening, which may have enriched the tested sample for more symptomatic or severe presentations. Third, the dataset does not include a population denominator, pre-pandemic comparison group, or post-restriction group, preventing true prevalence estimation and causal inference about NPIs. Fourth, the dataset did not include SARS-CoV-2 co-testing status, a validated disease-severity scoring instrument, radiological findings, or blood culture results, which limits assessment of SARS-CoV-2 co-infection and of relative bacterial versus viral contributions to severity. Fifth, because hospital admission/continued inpatient management was defined broadly to include continued observation beyond initial emergency evaluation (Methods), the low proportion of children with formal ward admission reflects this definition and should not be read as indicating a non-hospital-based sample. Finally, sparse-outcome regression for prognosis produced unstable estimates and was not suitable for primary interpretation; future analyses of similarly small, sparse-event pediatric ARI datasets may benefit from penalized regression approaches, such as Firth's correction, to reduce small-sample bias (29). Because mechanical ventilatory/noninvasive respiratory support was captured as a separate variable from formal admission status, these counts should not be assumed to be nested, and readers should refer to the Definitions subsection when comparing the two.

## 5 Conclusion

Among children with ARIs evaluated during the COVID-19 restriction period, most cases were mild and severe disease was infrequent. Lower oxygen saturation and longer symptom duration were the most clinically meaningful markers of greater severity and admission/continued inpatient management. Respiratory virus detection, especially rhinovirus, and pathogen co-detection were observed, but the study should be interpreted as a hospital-based clinical description rather than a denominator-based prevalence analysis. Larger multicenter studies with pre-pandemic, restriction-period, and post-restriction comparison groups are needed to clarify the long-term effects of pandemic control measures on pediatric respiratory pathogen epidemiology and clinical outcomes.

## Data Availability

The raw data supporting the conclusions of this article will be made available by the authors, without undue reservation.
